# The Application Value of Bronchoalveolar Lavage Fluid Metagenomic Next‐Generation Sequencing in Moderate‐to‐Severe Bronchiectasis

**DOI:** 10.1002/jcla.70156

**Published:** 2025-12-28

**Authors:** Jiachun Li, Qiuping Quan, Junshan Chen, Xiaoyun Jian, Weijie Zhan, Jingmin Wang, Rongbin Jiang

**Affiliations:** ^1^ Department of Respiratory Medicine The Eighth Clinical Medical College of Guangzhou University of Chinese Medicine Foshan Guangdong China; ^2^ Department of Respiratory Medicine Foshan Hospital of Traditional Chinese Medicine Foshan Guangdong China

**Keywords:** bronchoalveolar lavage fluid, conventional detection methods, metagenomic next‐generation sequencing, mixed infections, moderate‐to‐severe bronchiectasis

## Abstract

**Background:**

Bronchiectasis, a leading chronic airway disease, often worsens due to infections, making rapid pathogen detection crucial. This study aims to evaluate the diagnostic value of bronchoalveolar lavage fluid (BALF) metagenomic next‐generation sequencing (mNGS) in identifying pathogens in moderate‐to‐severe bronchiectasis and compare its advantages to conventional methods.

**Methods:**

Fifty‐two hospitalized patients initially diagnosed with moderate‐to‐severe bronchiectasis at Foshan Hospital of Traditional Chinese Medicine from May 2022 to March 2024 were enrolled. Clinical data and BALF samples were collected and subjected to both mNGS and conventional pathogen detection methods. The differences and concordance in pathogen distribution between mNGS and conventional methods, as well as their diagnostic performance, were compared.

**Results:**

The positive detection rate of pathogens by mNGS was significantly higher than that by conventional methods (*p* < 0.01). Both methods predominantly identified bacterial pathogens, with *Pseudomonas aeruginosa* being the most common bacterium and *Aspergillus fumigatus* the most frequent fungus. However, mNGS detected a broader range of pathogens and demonstrated superior sensitivity in identifying mixed infections (*p* < 0.01). The sensitivity of mNGS was 66% higher than that of conventional methods (*p* < 0.01), and the complete concordance rate between the two methods in double‐positive cases was 41.18%. Additionally, mNGS‐guided anti‐infection treatment significantly improves patient symptoms, reduces hospital stays, and lowers costs (*p* < 0.05).

**Conclusions:**

Compared with conventional methods, BALF mNGS demonstrates higher sensitivity, a greater positive detection rate, superior capability in identifying mixed infections, improved diagnostic performance, and a better guiding effect on anti‐infection treatment in moderate‐to‐severe bronchiectasis.

## Graphical abstract

This study analyzed the clinical data of 52 patients with moderate‐to‐severe bronchiectasis who were admitted to our hospital. It explored the diagnostic value of bronchoalveolar lavage fluid (BALF) metagenomic next‐generation sequencing (mNGS) in identifying infectious pathogens in patients with moderate‐to‐severe bronchiectasis, and clarified the advantages of mNGS over conventional pathogen detection methods.

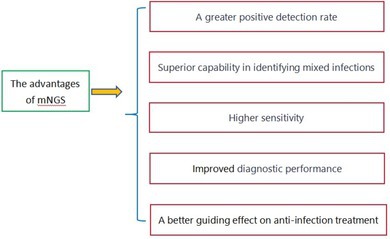

**Abbreviations**
BALF: bronchoalveolar lavage fluid
mNGS: metagenomic next‐generation sequencing

## 1 Introduction

Bronchiectasis is caused by recurrent suppurative infections induced by various etiologies. These infections lead to repeated damage or obstruction of the small and medium‐sized bronchi, ultimately disrupting the bronchial wall structure and resulting in abnormal and persistent dilation. Clinically, patients often present with chronic cough, excessive sputum production, intermittent hemoptysis, and varying degrees of dyspnea and respiratory failure [1]. In clinical practice, bronchiectasis has become the third most common chronic airway disease, and its prevalence is increasing worldwide [2]. Bronchiectasis imposes an increasingly heavy burden on global healthcare systems [3].

Bronchoalveolar lavage fluid (BALF) is a sample directly obtained from the lungs via bronchoalveolar lavage under fiberoptic bronchoscopy. This method is minimally invasive and offers a high pathogen detection rate [4]. Lower respiratory tract infections (LRTIs) are the primary cause of acute exacerbations in adults with bronchiectasis [5]. Therefore, identifying the causative pathogens in LRTIs is crucial for guiding bronchiectasis treatment. Conventional pathogen culture techniques often suffer from limitations such as long turnaround times and low positivity rates, making etiological diagnosis challenging. In contrast, metagenomic next‐generation sequencing (mNGS) is a novel nucleic acid detection technology capable of rapidly sequencing all nucleic acids in a specimen. By comparing the sequences with specialized databases, mNGS can accurately identify microbial species, enabling fast and precise pathogen detection. This approach demonstrates significant advantages in detecting rare or atypical pathogens [6]. Several studies [7, 8] have demonstrated the advantages of mNGS of BALF for diagnosing pulmonary pathogens, which also provides a theoretical basis for further research into its application in bronchiectasis‐related infections.

Performing mNGS on BALF provides a more comprehensive, rapid, and accurate representation of the microbial community in the lower respiratory tract, which is critical for identifying pathogens in moderate‐to‐severe bronchiectasis. However, reports on the application of mNGS for etiological diagnosis in such patients remain scarce, particularly studies utilizing BALF samples for mNGS testing. This study analyzed the clinical data of 52 hospitalized patients with moderate‐to‐severe bronchiectasis to evaluate the clinical utility of BALF‐based mNGS in pathogen detection. The findings are reported as follows.

## 2 Materials and Methods

### 2.1 General Data

We analyzed the clinical data of 52 hospitalized patients with moderate‐to‐severe bronchiectasis who underwent BALF mNGS testing alongside conventional pathogen detection (smear microscopy and culture) at Foshan Hospital of Traditional Chinese Medicine from May 2022 to March 2024. The diagnosis and severity grading of bronchiectasis were based on the 2021 Chinese Expert Consensus on the Diagnosis and Treatment of Adult Bronchiectasis [1] and the latest international consensus [5]. Severity was assessed using the Bronchiectasis Severity Index (BSI) [1]. The BSI scoring system incorporates eight variables: age, body mass index (BMI), FEV1 accounts for the percentage of the projected value, history of prior hospitalization for exacerbations, number of exacerbations in the previous year, Medical Research Council (MRC) dyspnea score, colonization by *Pseudomonas aeruginosa* and other microorganisms, and radiological involvement of ≥ 3 lobes or presence of cystic bronchiectasis. Each variable is assigned a specific score based on patient characteristics. The total score stratifies disease severity into three grades: mild (0–4 points), associated with a low risk of hospitalization and mortality; moderate (5–8 points), associated with a moderate risk; and severe (≥ 9 points), associated with a high risk. Among the 52 patients included, 20 were male and 32 were female, with an age range of 28–84 years (mean age 61.56 ± 12.89 years). In this study, BALF collection, mNGS testing, and conventional microbiological testing were performed following informed consent obtained from all patients or their legal guardians. The study protocol was approved by the Medical Ethics Committee of Foshan Hospital of Traditional Chinese Medicine and adhered to the principles of medical ethics (the ethics committee approval number: KY [2022] 219).

### 2.2 Methods

#### 2.2.1 Specimen Collection [4]

Preoperative preparations were completed, and informed consent was obtained. The target lung segment for lavage was determined based on clinical signs and imaging findings. After topical anesthesia with 2% lidocaine, a fiberoptic bronchoscope was advanced into the most severely affected segment. Sterile 37°C normal saline (25–50 mL per aliquot) was instilled and immediately aspirated under negative pressure (recovery rate: 30%–50%). The collected BALF was divided into two equal aliquots for mNGS and conventional testing.

#### 2.2.2 mNGS Pathogen Detection [9]

Collect qualified samples according to the above steps, extract nucleic acids for high‐throughput sequencing, and conduct bioinformatics analysis on the nucleic acid sequences of microorganisms in the samples to obtain the test results.

#### 2.2.3 Conventional Pathogen Detection

This primarily includes bacterial culture and smear, fungal culture, and acid‐fast bacillus (AFB) smear testing for *Mycobacterium tuberculosis* . (1) Professional technicians strictly followed the procedures outlined in the *National Clinical Laboratory Operating Procedures* (4th edition) [10], using culture media such as blood agar, MacConkey agar, and chocolate agar for cultivation. (2) In the laboratory, Gram staining and acid‐fast staining were performed on BALF, followed by microscopic examination of smears for bacteria, fungi, and *M. tuberculosis* , respectively.

#### 2.2.4 Observation Indicators

The detection results of conventional methods and mNGS were observed, and the performance of BALF conventional testing versus mNGS was compared (including pathogen detection rate, species distribution, and concordance between the two methods).

### 2.3 Criteria for “Confirmed” Pathogen Diagnosis Based on Etiological Detection and Clinical Comprehensive Analysis

Patients were classified into the confirmed pathogen group if they met any of the following criteria [11]: (1) Concordant results between conventional culture and mNGS; (2) Positive conventional culture with clinical correlation or responsive to targeted therapy; (3) Positive mNGS with clinical correlation or responsive to targeted therapy; (4) Discordant results between conventional culture and mNGS, requiring comprehensive analysis incorporating clinical judgment and treatment response.

Patients who did not meet the above criteria were classified into the unconfirmed pathogen group.

### 2.4 Statistical Analysis

All patients' data were systematically compiled and entered into an Excel database. Statistical analysis was performed using SPSS 22.0. Continuous variables were expressed as mean ± standard deviation ($\bar{x}$ ± *S*). For normally distributed data, comparisons were made using the independent samples *t*‐test. For non‐normally distributed data, the Mann–Whitney *U* test (rank‐sum test) was applied. Categorical variables were presented as counts or percentages and analyzed using the Chi‐squared (*χ* ^2^) test, McNemar's test, or Fisher's exact test, as appropriate. A two‐tailed *p*‐value < 0.05 was considered statistically significant, while *p* < 0.01 indicated high statistical significance.

Sample size power analysis: To ensure the reliability of the current sample size (*n* = 52) for the main conclusion, we conducted a statistical power analysis during the study design stage (*α* = 0.05, test power = 80%). Sensitivity comparison: Set the sensitivity benchmark value of the traditional method at 30%, and expect the sensitivity of mNGS to increase to ≥ 60% (minimum clinical significance difference = 30%). Using the paired design (McNemar test) for estimation, the total sample size required to meet the test efficacy (i.e., the number of cases that can provide a sufficient number of inconsistent pairs) is at least 47 cases. Comparison of the detection rate of mixed infections: The expected detection rate of mNGS is 40%, while that of the traditional method is 10% (a difference of 30%). Also using the paired design (McNemar test) for estimation, the total sample size required to meet the test efficacy is at least 37 cases. A total of 52 patients were ultimately included in this study, and the sample size met the minimum values of the above two estimates (47 cases and 37 cases).

## 3 Results

### 3.1 Baseline Characteristics of Patients

A total of 52 eligible patients were enrolled in this study, comprising 20 males (38.46%) and 32 females (61.54%), with a mean age of 61.56 ± 12.89 years. Details are presented in Table 1. BALF samples were collected from all 52 patients with moderate‐to‐severe bronchiectasis and simultaneously subjected to mNGS and paired conventional testing.

**TABLE 1:** Baseline characteristics of the patients.

Characteristic	Statistical value
Age, years (Mean ± SD)	61.56 ± 12.89
Male [*n* (%)]	20 (38.46%)
Disease duration, months (mean ± SD)	21.75 ± 10.73
Number of involved lung lobes [*n* (%)]	
1 lobe	[0 (0.00%)]
2 lobes	[2 (3.85%)]
3 lobes	[32 (61.54%)]
4 lobes	[15 (28.85%)]
5 lobes	[3 (5.77%)]
Main symptoms [*n* (%)]	
Cough	[1 (1.92%)]
Cough; expectoration	[32 (61.54%)]
Cough; expectoration; hemoptysis	[8 (15.38%)]
Cough; expectoration; shortness of breath	[6 (11.54%)]
Cough; expectoration; shortness of breath; hemoptysis	[5 (9.62%)]
Smoking history [*n* (%)]	22 (42.31%)
Comorbid asthma [*n* (%)]	6 (11.54%)
Comorbid COPD [*n* (%)]	7 (13.46%)
Comorbid diabetes mellitus [*n* (%)]	5 (9.62%)
Comorbid hypertension [*n* (%)]	10 (19.23%)
Comorbid rhinosinusitis [*n* (%)]	8 (15.38%)
History of tuberculosis [*n* (%)]	2 (3.85%)

### 3.2 Comparison Between mNGS and Conventional Detection Methods

In accordance with the positive criteria for mNGS and conventional detection methods [12], although mNGS identified certain microorganisms (such as *Enterococcus faecium* , *Enterococcus avium* , and *Candida glabrata*), these were excluded from positive results because they had sequence counts < 50 and lacked clinical correlation. These were consequently considered as potential colonizing microbiota rather than pathogenic microorganisms. The final comparative results are presented below.

#### 3.2.1 Comparison of Pathogen Detection Rates Between mNGS and Conventional Methods

Among the 52 patients with moderate‐to‐severe bronchiectasis, mNGS yielded positive results in 50 cases (96.15% positivity rate) and negative results in 2 cases. In contrast, conventional microbiological methods detected pathogens in only 17 cases (32.69% positivity rate) with negative results in 35 cases. The pathogen detection rate of mNGS was significantly higher than that of conventional methods (*χ* ^2^ = 31.03, *p* < 0.001).

#### 3.2.2 Pathogen Distribution Patterns Between mNGS and Conventional Methods

**FIGURE 1:**
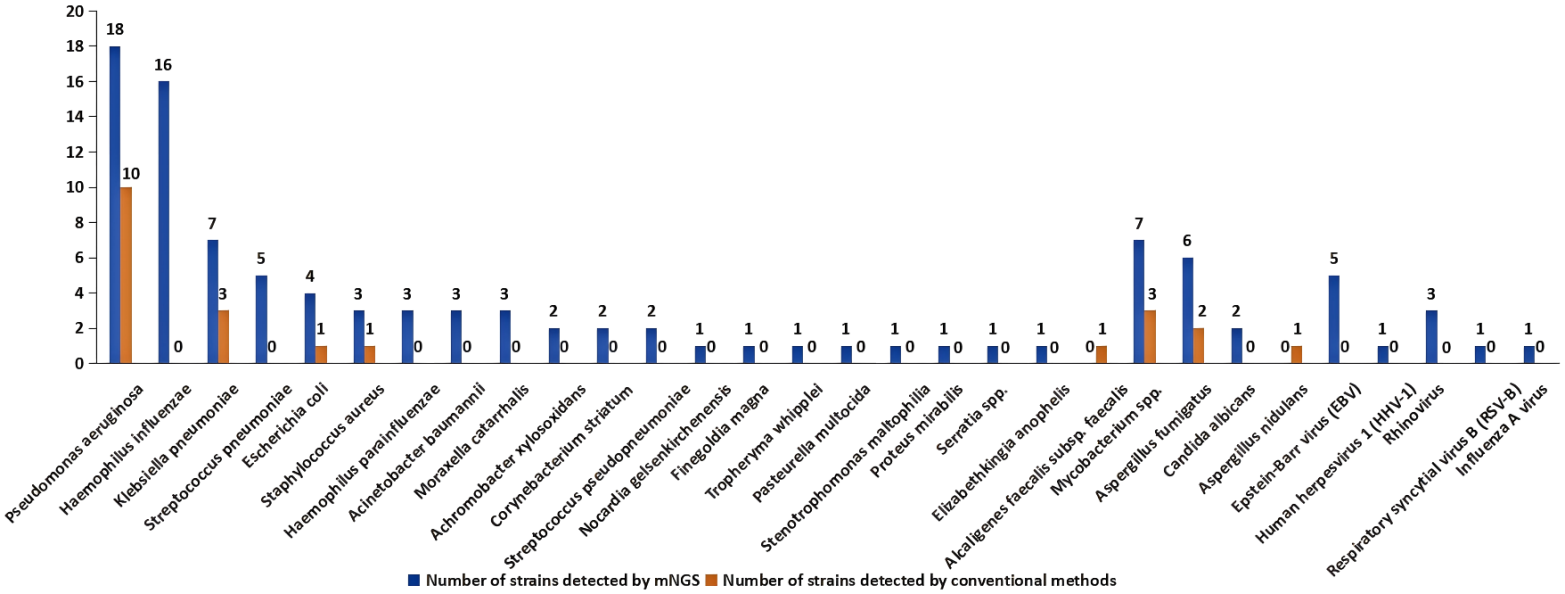
Distribution of pathogens detected by mNGS and conventional methods in bronchoalveolar lavage fluid.

Among the 50 mNGS‐positive BALF samples, a total of 102 pathogenic strains were identified, comprising 76 bacterial strains, 8 fungal strains, 6 DNA viral strains, 5 RNA viral strains, and 7 mycobacterial strains (including 2 *M. tuberculosis* , 2 *M. avium* , 2 *M. abscessus* , and 1 *M. intracellulare* ). In contrast, conventional methods detected pathogens in 17 cases, with a total of 22 pathogenic strains identified, comprising 16 bacterial strains, 3 fungal strains, and 3 mycobacterial strains (all acid‐fast bacilli). No viral strains were detected (detailed distribution is shown in Figure 1).

Both mNGS and conventional methods mainly detected bacterial pathogens. mNGS identified 20 types of bacteria, among which *P. aeruginosa* was the most frequently detected (18 strains, 23.68%), followed by *Haemophilus influenzae* (16 strains, 21.05%). Conventional detection methods identified five types of bacteria, with *P. aeruginosa* being the most frequently detected (10 strains, 62.50%). mNGS identified two types of fungi and four types of mycobacteria, with *A. fumigatus* being the most frequently detected among the fungal pathogens (6 strains, 75%). Conventional detection methods identified two types of fungi and one type of mycobacteria. mNGS identified five types of viruses, including two DNA viruses and three RNA viruses. Among the DNA viruses, Epstein–Barr virus was the most common, and among the RNA viruses, rhinovirus was the most common. It can be seen that there are certain differences between mNGS and conventional methods in pathogen detection. Conventional detection methods may miss many pathogens, while mNGS can detect more pathogens and has a broader coverage. Since traditional methods (smear and culture) are difficult to detect viruses, mNGS has a unique advantage in virus detection. Therefore, when considering the possibility of viral infection, mNGS detection can be considered [13].

#### 3.2.3 Comparison of mNGS and Conventional Methods in Detecting Mixed Infections

**FIGURE 2:**
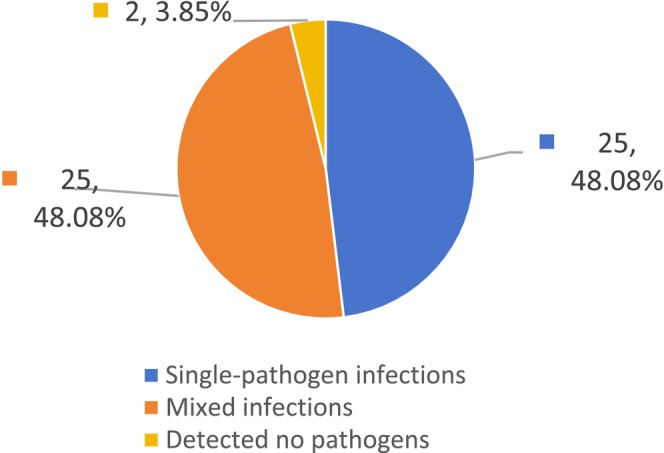
Proportion of mixed infections detected by mNGS.

**FIGURE 3:**
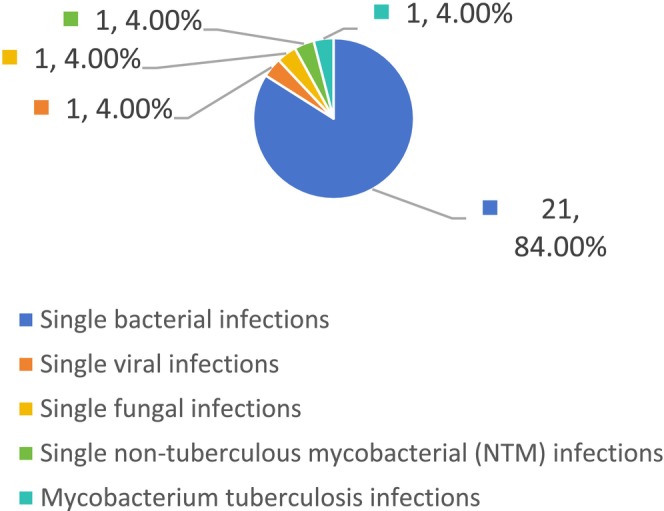
Categories and proportions of single‐pathogen infections detected by mNGS.

**FIGURE 4:**
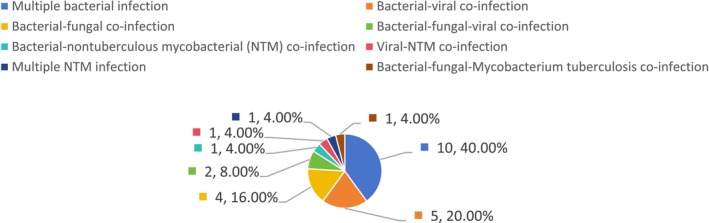
Categories and proportions of mixed infections detected by mNGS.

Among the 52 patients, BALF mNGS detected no pathogens in two cases (3.85%), while single‐pathogen infections were identified in 25 cases (48.08%). Of these, single bacterial infections accounted for 21 cases (84%), whereas single viral, fungal, non‐tuberculous mycobacterial (NTM), and *M. tuberculosis* infections were each detected in 1 case (4% each). Mixed infections were identified in 25 patients (48.08%), comprising 10 cases (40.00%) of multiple bacterial infection (≥ 2 bacterial species), 5 cases (20.00%) of bacterial‐viral co‐infection, 4 cases (16.00%) of bacterial‐fungal co‐infection, 2 cases (8.00%) of bacterial‐fungal‐viral co‐infection, and one case each (4.00%) of bacterial‐nontuberculous mycobacterial (NTM) co‐infection, viral‐NTM co‐infection, multiple NTM infection (≥ 2 NTM species), and bacterial‐fungal‐ *M. tuberculosis* co‐infection (see Figures 2, 3, and 4).

**FIGURE 5:**
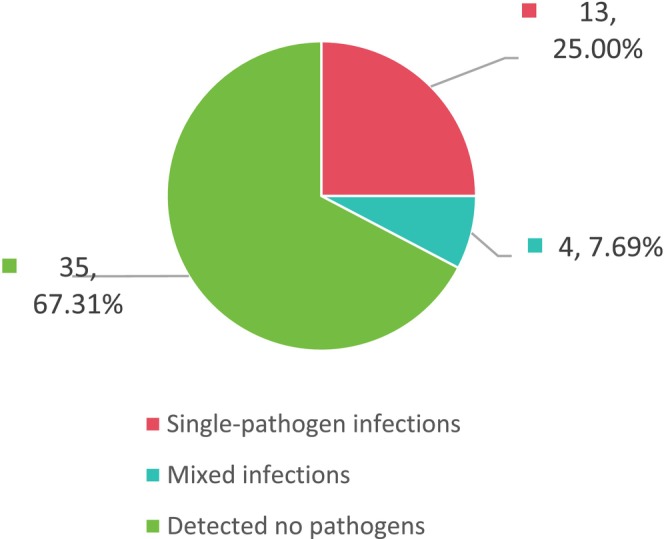
Proportion of mixed infections detected by conventional methods.

**FIGURE 6:**
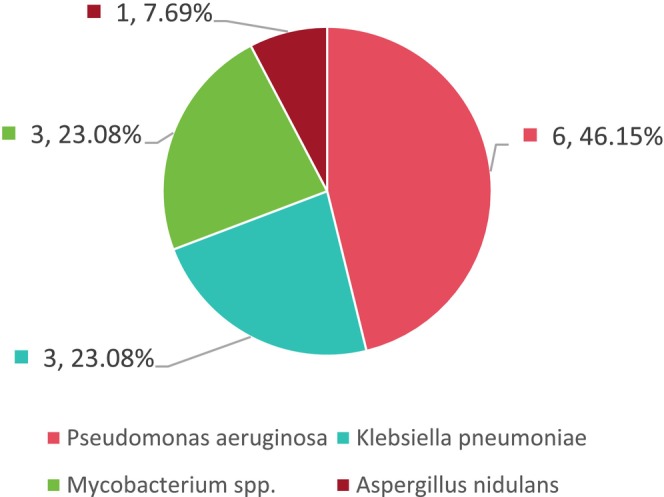
Categories and proportions of single‐pathogen infections detected by conventional methods.

**FIGURE 7:**
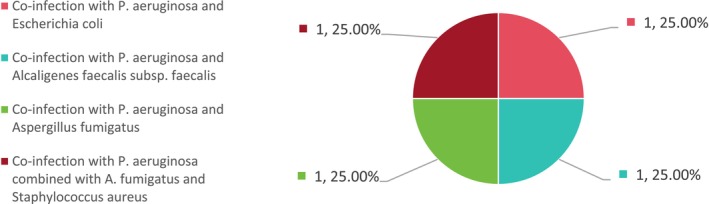
Composition and distribution of mixed infections detected by conventional methods.

Results of conventional testing showed no pathogens detected in 35 cases (67.31%), single‐pathogen infections in 13 cases (25%), and mixed infections in only four cases (7.69%) (see Figure 5). Among patients with single‐pathogen infections, *P. aeruginosa* was identified in six cases (46.15%), *Klebsiella pneumoniae* in three cases (23.08%), Mycobacterium spp. in three cases (23.08%), and *Aspergillus nidulans* in one case (7.69%) (see Figure 6). The mixed infection cases included co‐infection with *P. aeruginosa* and *Escherichia coli* (1 case, 25%), *P. aeruginosa* and *Alcaligenes faecalis* subsp. faecalis (1 case, 25%), *P. aeruginosa* and *A. fumigatus* (1 case, 25%), and *P. aeruginosa* combined with *A. fumigatus* and *Staphylococcus aureus* (1 case, 25%) (see Figure 7).

These results demonstrate that among the 52 patients with moderate‐to‐severe bronchiectasis, mNGS detected significantly more mixed infections than conventional methods (48.08% vs. 7.69%, *p* < 0.01). Among single‐pathogen infections identified by mNGS, bacterial infections were most common, with *H. influenzae* being the predominant species (8 cases, 38.10%), followed by *P. aeruginosa* (6 cases, 28.57%). In mixed infections, polymicrobial bacterial co‐infections accounted for the highest proportion. Among these mixed infection cases, *P. aeruginosa* was the most prevalent pathogen (with the highest sequence count) (11/25, 44% [95% CI: 25.6%–64.2%]), followed by *H. influenzae* (5/25, 20% [95% CI: 8.9%–39.1%]). In conventional testing, *P. aeruginosa* was the most frequently detected pathogen in both single‐pathogen and mixed infections.

### 3.3 Interpretation of Pathogens Based on mNGS, Conventional Detection Methods, and Clinical Data

The mNGS results showed 50 positive and 2 negative cases, while conventional detection methods yielded 17 positive and 35 negative cases. The definitive pathogen diagnosis (determined through comprehensive analysis integrating mNGS, conventional detection results, and clinical patient data) identified 50 positive and 2 negative cases. Among the 50 confirmed pathogens, 33 were mNGS‐positive but conventional detection‐negative, while 0 were conventional detection‐positive but mNGS‐negative. Pathogens detected in the 50 mNGS‐positive patients were ultimately confirmed as definitive pathogens, and pathogens detected in the 17 conventional detection‐positive patients were also confirmed as definitive pathogens. Among the two non‐confirmed pathogen cases, both mNGS and conventional detection results were negative (see Table 2).

**TABLE 2:** The results of pathogen interpretation based on mNGS, conventional detection methods and clinical data.

	Positive	Negative
mNGS	50	2
Conventional methods	17	35
Confirmed pathogens	50	2

### 3.4 Comparison of Sensitivity and Concordance Between mNGS and Conventional Detection Methods

BALF mNGS demonstrated a 66% higher sensitivity than conventional detection (100% vs. 34%, *p* < 0.01). Seventeen cases (32.69%) were positive by both mNGS and conventional methods. Two cases (3.85%) were negative by both methods. Thirty‐three cases (63.46%) were positive by mNGS but negative by conventional detection. Among the 17 cases with dual positivity (both mNGS and conventional methods), 7 cases (41.18%) showed complete concordance between mNGS and conventional results. Ten cases exhibited partial concordance, with mNGS detecting additional pathogens compared to conventional methods. Thus, the complete concordance rate between the two methods in dual‐positive cases was 41.18%.

### 3.5 Effectiveness of Anti‐Infective Therapy Guided by mNGS Detection Results

Among the 50 patients with moderate‐to‐severe bronchiectasis who tested positive by mNGS, anti‐infective therapy guided by the mNGS results led to significant improvement in clinical symptoms, signs, and infection biomarkers compared to baseline. Furthermore, the average length of hospital stay and average hospitalization cost for these 50 patients were 7.85 days and ¥9932.56, respectively. For comparison, we analyzed data from another fifty patients with moderate‐to‐severe bronchiectasis hospitalized at Foshan Hospital of Traditional Chinese Medicine who received empiric anti‐infective therapy or therapy guided solely by conventional detection methods (without mNGS). There was no statistically significant difference in baseline data such as age, gender, and underlying diseases between the two groups of patients. The average length of hospital stay and average hospitalization cost for the latter group were significantly higher, at 9.36 days and ¥11,548.67, respectively. These findings demonstrate that anti‐infective therapy guided by mNGS detection results is more effective in improving patients' clinical manifestations and significantly reduces both the average length of hospital stay and average hospitalization cost (*p* < 0.05).

### 3.6 Sample Size Power Validation Results

Based on the preset parameters (*α* = 0.05, power = 80%): (1) Sensitivity comparison: The actual detection sensitivity of mNGS (100%) was 66% higher than that of the conventional methods (34%) (*p* < 0.01). The power analysis indicated that the current sample size could detect a sensitivity difference of ≥ 30% (minimum required: 47 cases; actual *n* = 52 in this study). (2) Detection rate of mixed infections: The detection rate of mNGS (48.08%) was significantly higher than that of the conventional methods (7.69%) (*p* < 0.01), and the power analysis supported the detection of a rate difference of ≥ 30% (minimum required: 37 cases). (3) Limitations of subgroup analysis: In the mNGS mixed‐infection subgroup, the 95% CI for *P. aeruginosa* ‐dominant infections (*n* = 11) was 25.6%–64.2%, and the 95% CI for *H. influenzae* ‐dominant infections (*n* = 5) was 8.9%–39.1%. Due to the small sample size, precision of these estimates was limited.

## 4 Discussion

Early pathogen diagnosis is of great significance for the rational use of anti‐infective drugs in patients with moderate‐to‐severe bronchiectasis. Conventional diagnostic techniques, such as smear microscopy and microbial culture, are time‐consuming, have a narrow detection range, and exhibit low positivity rates, often failing to meet the diagnostic needs of these patients. With advances in molecular biology, the clinical value of mNGS has gained increasing recognition, particularly for detecting rare, atypical, or slow‐growing microorganisms, identifying pathogens undetectable by current methods, and aiding in cases who do not respond to standardized anti‐infection treatment [14]. It is necessary to emphasize that in the interpretation of mNGS reports for bronchiectasis infection, we need to combine the actual clinical situation to rule out respiratory colonizing pathogens or contaminating bacteria.

This study analyzed the detection results and pathogen profiles of BALF mNGS and conventional pathogen testing in 52 patients with moderate‐to‐severe bronchiectasis, comparing the discrepancies and concordance between the two methods. First, mNGS demonstrated a significantly higher pathogen detection rate than conventional methods (96.15% vs. 32.69%, *p* < 0.01). Second, while BALF mNGS and conventional testing showed consistency in pathogen distribution (both predominantly detecting bacteria, with *P. aeruginosa* being the most common bacterial species and *A. fumigatus* the most frequent fungus), mNGS exhibited broader coverage, detecting a wider range of pathogens, including viruses, with unique advantages. Furthermore, mNGS outperformed conventional methods in identifying mixed infections (48.08% vs. 7.69%, *p* < 0.01), suggesting its utility when viral or polymicrobial infections are suspected.

Notably, mNGS exhibited 66% higher sensitivity than conventional testing (100% vs. 34%, *p* < 0.01), demonstrating superior diagnostic performance. Among the 17 cases positive by both methods, the complete concordance rate was 41.18%. In the 10 partially concordant cases, mNGS provided more comprehensive results, detecting additional pathogens missed by conventional methods. Moreover, among the 35 cases negative by conventional testing, 33 were positive by mNGS. These findings suggest that mNGS can identify a greater number of pathogens, playing a significant role in the etiological diagnosis of moderate‐to‐severe bronchiectasis. Under appropriate conditions, mNGS may replace conventional methods to guide clinical diagnosis and precision treatment [13].

Our study indicates that while BALF mNGS and conventional testing share overlapping pathogen profiles, mNGS offers higher sensitivity, is less affected by prior antibiotic exposure [15], and detects more pathogens. It is particularly advantageous in identifying rare pathogens such as *Nocardia* and nontuberculous mycobacteria, enabling rapid diagnosis and precise anti‐infection treatment. Additionally, mNGS demonstrates superior performance in viral detection. Therefore, early identification of rare and pathogenic viruses through BALF mNGS testing holds significant clinical importance, enabling timely and appropriate medical decision‐making [16]. Since mNGS can simultaneously detect multiple pathogens in a single test, it is especially beneficial for patients with mixed infections, effectively preventing the abuse of anti‐infective drugs and the occurrence of drug resistance [17].

Liu [18] conducted mNGS and conventional pathogen detection on bronchoalveolar lavage fluid samples from 170 bronchiectasis patients, demonstrating significantly higher pathogen detection rates with mNGS compared to culture methods (*p* < 0.05). In a retrospective analysis of 67 bronchiectasis cases, Shen et al. [3] collected clinical baseline data and pathogen reports, revealing that mNGS identified substantially greater pathogen diversity and quantity than conventional culture. The most frequently detected bacterial pathogens were *P. aeruginosa* and *H. influenzae* , while *A. fumigatus* predominated among fungal pathogenic bacteria. The results of this study are consistent with the research of scholars such as Liu and Shen et al. mentioned above. Moreover, this study further verified the significant clinical value of mNGS in guiding anti‐infection treatment for patients with moderate‐to‐severe bronchiectasis.

While BALF mNGS in this study demonstrated an exceptionally high pathogen‐positive detection rate (96.15%), we fully recognize that this high sensitivity necessitates careful interpretation of the specificity of the results, particularly in distinguishing true pathogens from oral or environmental contaminants and respiratory colonizing bacteria. To ensure diagnostic accuracy, we implemented a series of comprehensive measures. First, during specimen collection, all bronchoalveolar lavage procedures strictly adhered to aseptic principles and were performed by experienced pulmonologists under bronchoscopic visualization, with the scope wedged into the target diseased lung segment to minimize contamination from upper respiratory secretions and oropharyngeal colonizing flora. Second, during the bioinformatic analysis and clinical interpretation phase, we did not rely solely on the detection or non‐detection of microorganisms but introduced a quantitative threshold for sequence counts (set at 50 in this study) as an auxiliary tool for the preliminary screening of potential pathogens. This threshold was not an absolute criterion but an empirical cutoff established based on relevant literature and retrospective analysis of a large number of clinical samples, aiming to filter out those microorganisms with extremely low sequence counts, which were more likely to represent contaminating microorganisms or colonizing microbes. For instance, microorganisms such as *E. faecium* , *E. avium* , and *C. glabrata* detected by mNGS in this study had read counts below this threshold and were inconsistent with the patients' concurrent clinical manifestations, imaging features, and levels of other inflammatory biomarkers (e.g., PCT, CRP). Therefore, after comprehensive assessment, they were considered potential colonizing or contaminating microorganisms and were not included in the final list of pathogenic pathogens.

However, it must be emphasized that a sequence count threshold is not a “gold standard” for differentiating colonization from infection. The core of clinical interpretation lies in the deep integration of mNGS results with the patient's specific clinical context. This includes evaluating whether the detected microorganism is a common pathogen for pulmonary infection in such patients, its relative abundance within the detected microbial community, its correlation with imaging signs of acute infection, and the patient's response to empirical antimicrobial therapy targeted against that pathogen. In the future, developing multi‐parameter models that integrate microbial sequence counts, host immune response markers (such as host‐directed mRNA expression profiles), and clinical scoring systems will be a crucial direction for further enhancing the diagnostic specificity of mNGS and achieving precise discrimination between pathogens and colonizing or contaminating microorganisms.

This study verified the reliability of 52 samples to the core conclusion (the sensitivity and the advantage in detecting mixed infections of mNGS vs. conventional methods) through statistical power analysis. However, subgroup analysis (such as the pathogen spectrum distribution of mixed infections) has the following limitations due to insufficient sample size: small sample subgroups (e.g., only one case of mixed infection with multiple nontuberculous mycobacteria) cannot be statistically inferred; the confidence intervals of key subgroups (e.g., mixed infections dominated by *P. aeruginosa* ) are relatively wide, and the sample size needs to be expanded to improve accuracy. Future multi‐center studies are needed to further verify subgroup differences (such as the clinical significance of different pathogen combinations).

Our study demonstrates that mNGS, as an unbiased, culture‐independent detection method with high accuracy [19], plays a crucial role in the diagnosis and treatment of moderate‐to‐severe bronchiectasis. However, several limitations should be acknowledged: (1) Clinical samples often contain multiple pathogens, and the current lack of authoritative, standardized interpretation criteria increases the difficulty of result analysis [3]; (2) Nucleic acid contamination from human normal microbiota or other sources may complicate the differentiation between true pathogens and colonizing microorganisms [20]; (3) The relatively high cost restricts its widespread clinical application; (4) This study still lacks an exploration of the functions of mNGS in detecting drug resistance genes and virulence factors; (5) The limited sample size in this study necessitates additional prospective, multicenter data to fully validate the clinical advantages of BALF mNGS testing.

## 5 Conclusions

In summary, BALF mNGS enables rapid and accurate pathogen detection and identification, establishing a foundation for precise diagnosis and personalized treatment. Furthermore, mNGS demonstrates significant advantages in diagnosing polymicrobial infections in patients with moderate‐to‐severe bronchiectasis, representing a promising diagnostic technology for clinical infectious diseases. We recommend mNGS as an important supplementary diagnostic tool for pathogen identification. Anti‐infection treatment guided by the BALF mNGS detection results can not only significantly improve the clinical manifestations of patients but also significantly reduce the length of hospital stay and hospitalization costs of patients. In the future, prospective and multi‐center studies on drug resistance gene detection need to be combined to further verify the clinical value of its treatment success rate. As technological and economic advancements continue to address its current limitations, the clinical application of mNGS is expected to become increasingly widespread.

## Author Contributions

Jiachun Li and Qiuping Quan contributed equally to this work and should be considered co‐first authors. Conception, design or planning of the study: Jiachun Li and Qiuping Quan. Interpretation of the results: Jiachun Li, Qiuping Quan, Junshan Chen, Xiaoyun Jian, Weijie Zhan, Jingmin Wang and Rongbin Jiang. Drafting of the manuscript: Jiachun Li and Qiuping Quan. Critically reviewing or revising the manuscript for important intellectual content: Jiachun Li, Qiuping Quan, Junshan Chen, Xiaoyun Jian, Weijie Zhan, Jingmin Wang, and Rongbin Jiang. All authors approve the final version to be published and agree to be accountable for all aspects of the work.

## Funding

Self‐funded category: 2022 Foshan Medical Science and Technology Research Project (2220001004976).

## Ethics Statement

This study strictly adhered to the relevant provisions of the Declaration of Helsinki and was approved by the Ethics Committee of Foshan Hospital of Traditional Chinese Medicine.

## Consent

All participants were given informed consent and had the right to withdraw from the study at any time. Their names and other confidential information were protected. No harm was caused to the participants during the study.

## Conflicts of Interest

The authors declare no conflicts of interest.

## Data Availability

The data that support the findings of this study are available from the corresponding author upon reasonable request.
